# Production performance of Charolais crossbred steers fed total mixed ration containing a high level of dried cassava top

**DOI:** 10.5455/javar.2023.j704

**Published:** 2023-09-30

**Authors:** Ekkapan Inngarm, Ruangyote Pilajun, Kungwan Thummasaeng, Areerat Lunpha, Sophany Morm

**Affiliations:** 1 Department of Animal Science, Faculty of Agriculture, Ubon Ratchathani University, Ubon Ratchathani, Thailand; 2 Department of Animal Science, Faculty of Agriculture and Food Processing, National University of Battambang, Battambang, Cambodia

**Keywords:** Cassava top, carcass characteristic, economic return, Charolais crossbred steers

## Abstract

**Objective:**

The objectives were to determine the appropriate level of dried cassava top (DCT) in total mixed ration (TMR) based on production performance and carcass characteristics of fattening Charolais crossbred steers.

**Materials and Methods:**

Fifteen fattening Charolais crossbred steers were randomly assigned to a randomized complete block design with initial body weight to receive three treatments, including without DCT, 15% DCT, and 30% DCT in TMR, on a dry matter (DM) basis. The production trial lasted 120 days; then, the fattened steers were slaughtered to study the carcass characteristics.

**Results:**

The *in vitro* gas production from the immediately soluble fraction of TMR containing 30% DCT was higher than the others (*p *< 0.05). However, *in vitro* DM and organic matter degradability were not significantly different among treatments. Feed intake, final weight, feed cost per gain, and carcass characteristics such as warm carcass percentage, marbling score, and loin eye area of feedlot steers were not affected by the inclusion of DCT in TMR. Although steers fed TMR containing 15% DCT had body weight gain, average daily gain, and feed conversion ratio lower than the control (*p *< 0.05); however, the use of DCT at 30% DM in TMR reduced the feed cost of feedlot beef production (*p *< 0.05).

**Conclusion:**

Using local ingredients such as cassava tops can increase profit margins for farmers without sacrificing product quality, but they must closely look at growth performance.

## Introduction

Farmers are interested in beef cattle fattening since premium meat consumption has recently increased in the country. Moreover, beef cattle are easy to manage and generate additional income for farmers [1]. The people of Thailand prefer beef for their daily needs. In addition, high market demand for the finished cattle divided them into two groups. Short-time fattening starts with mature or old cattle for about 4 months to supply the traditional market, while premium beef has to be produced from growing cattle (~2 years old) for >12 months until nearly 4 years old [2]. The fattening cattle were separated into two feeding systems: feeding and total mixed ration (TMR). The homogenized feed made from completely mixed ingredients formulated with specific nutrient contents was used later. It can improve the rumen condition, especially by reducing rumen pH fluctuation and creating a more favorable environment for rumen microbes than separated feeds [3,4]. However, although fattening cattle can generate income for the farmers, feedstuff prices increase continuously [5], so the cost of fattening cattle production also rises. Using agricultural by-products to replace costly raw materials is one way to decrease feed costs.

Cassava is a major economic crop in several countries. After harvest, it was found that there were about 1.88–3.13 tons/hectare of cassava tops (CT), unutilized waste, remaining [6]. These CT, green stem, petiole, and leaves collected at the time of cassava root harvesting are interesting raw materials, containing varying levels of crude protein (CP), reported as 21.87%–40% [7–9], while CT-fermented with live yeast has between 20.76 and 21.55 [7,8]. In addition, 10% dried CT (DCT) fermented cassava pulp-activated *Saccharomyces cerevisiae* can improve CP content, *in vitro* dry matter (DM) disappearance (IVDMD), and gas production from the immediately soluble fraction [10]. In addition, ruminants will be toxic when they consume fresh cassava leaves containing high hydrocyanic acid (HCN) without processing. Sun-drying effectively reduces HCN in cassava leaves [11]. Whereas condensed tannin has the potential to decline the gastro-intestinal parasitic eggs effectively [12] by diminution in the worm burden and/or a reduction in the fecundity of female worms [13] and the small intestine by increasing by-pass protein and amino acids [14]. A varied study supplemented with dry cassava leaves could improve feed intake, milk fat, and milk yield among 3.5% of fat-corrected milk when provided for lactating cows between 115 and 120 days at 1.7 kg/head/day [15]. Cassava hay is a protein source that could replace soybean meal concentrate for decreased feed costs and increased income over commercial feed [16]. Moreover, Kounnavongsa et al. [17] found that goats fed cassava foliage (fresh cassava stem, petiole, and leaves harvested at the early stage) had 35% higher weight gain and 36% better feed conversion ratio (FCR) in contrast with the sun-dried form based on Gamba grass or sugar cane stalk. In addition, cassava leaves can decrease the protozoa population in the rumen. However, studies on using CT as a protein source in TMR still need to be improved, especially in high-quality fattening cattle that require specific nutrient concentrations and high-quality feed.

Therefore, the objectives of our experiments were to determine the appropriate level of DCT, an inexpensive and sustainable animal feed, in TMR based on *in vitro* gas production, production performance, and carcass characteristics of fattening Charolais crossbred steers.

## Materials and Methods

### Ethical statement

The authors confirm adherence to the ethical policies of the journal and that all animal procedures were approved by the Animal Care and Use for Scientific Purposes Committee, Ubon Ratchathani University, Warinchamrap, Ubon Ratchathani, Thailand [34190].

### Experimental design

The experiment was carried out at a private feedlot cattle farm located in Thung Thoeng Subdistrict, Det Udom District, Ubon Ratchathani Province, Thailand (14.82250, 104.86082). CTs were collected from cassava fields before cassava root harvesting from October to November 2019. The cassava stem was broken between green and brown color, then CT was cassava leaves and petioles and a green stem. Chopped CT, 2–3 cm, were dried under the sun for 2–3 days to have moisture lower than 15% before storing in a plastic bag. After animal adaptation, the experiment lasted 4 months, from December 2019 to March 2020. Treatments included the inclusion of DCT in TMR, including 0% (control), 15%, and 30% DM. Feed formulation was followed as recommended by the Working Committee of the Thai Feeding Standard for Ruminants [18] to meet the average daily gain (ADG) of 1.0 kg/day. Feed samples were taken for chemical analysis of DM, CP, ether extract (EE), and total ash using proximate analysis [19], neutral detergent fiber (NDF), and acid detergent fiber (ADF) according to Van Soest et al. [20], without an amylase and inclusive of residual ash. Organic matter (OM) was calculated from total ash, while nonstructural carbohydrate was calculated from other nutrient percentages subtracted from 100%. In addition, total digestible nutrient (TDN) (%) was estimated from the TDN content of each feed ingredient. Ingredients and chemical compositions of experimental diets are presented in Table 1.

### In vitro gas production and degradability of experimental diets

A 14% CP of concentrate fed three >87.5% Holstein Friesian heifers at 0.5% BW and corn silage on an *ad libitum* basis. These heifers were given 500 ml of rumen fluid by stomach tube with a vacuum pump and filtered through four layers of cheesecloth into pre-warmed thermos flasks. The rumen fluid from three heifers was mixed, then artificial saliva was added at a 2:1 ratio of artificial saliva to rumen fluid [21]. Samples of DCT, corn silage, and experimental diets containing approximately 250 mg (milled through a 1.0 mm sieve) were weighed into 60 ml bottles and pre-warmed in a hot air oven at 39°C for 24 h before being filled with 35 ml of rumen inoculum mixture. The bottle, which contained samples and inoculums, was then closed with a rubber lid and aluminum cap. The gas production was recorded before incubation (0 h) and after 1, 2, 4, 6, 8, 12, 16, 20, 24, 30, 36, 48, 60, 72, and 96 h. Gas volume was detected by using a glass syringe connected with a rubber tube and a 22-gauge needle through a rubber lid. Cumulative gas production data were fitted into the model of Ørskov and McDonald [22] by the NEWAY computer package program:

*y* = *a* + *b *(1–*e^−ct^*)

where *a* is the gas production from the soluble fraction, *b* is the gas production from the insoluble fraction, *c* is the gas production rate, *t* is incubation time, (*a* + *b*) is the potential extent of gas production, and *y* is the gas produced at a time “*t*.” Digestible OM (DOM, %) and metabolizable energy (ME, MJ/kg DM) were calculated by using the equation of Menke and Steingass [21] as follows:

DOM, % = 0.9991 GP + 0.595 CP + 0.181 CA + 9

**Table 1.** Ingredients and chemical composition in experimental diets (DM basis).

Ingredients	Corn silage	DCT	Ctrl	15% DCT	30% DCT
Corn silage			40.0	40.0	40.0
DCT			0.0	15.0	30.0
Cassava chip			39.5	30.3	21.4
Palm kernel meal			5.00	5.00	5.00
Soybean meal			13.2	7.40	1.30
Molasses			1.00	1.00	1.00
Urea			0.50	0.50	0.50
Sulfur			0.05	0.05	0.05
Premix^a^			0.25	0.25	0.25
Salt			0.50	0.50	0.50
Chemical composition					
DM	39.0	93.9	58.1	62.0	58.4
OM, %DM	95.3	90.4	94.7	93.2	91.6
CP, %DM	6.91	15.0	11.4	11.6	12.8
EE, %DM	4.72	6.47	2.61	3.59	4.50
NSC, %DM	23.1	15.0	47.2	38.3	28.1
NDF, %DM	60.6	54.0	33.5	39.7	46.2
ADF, %DM	22.4	37.8	14.6	19.2	23.8
TDN, %DM	56.0	60.0	69.0	67.4	65.7
Price, GBP/kg	0.042	0.071	0.21	0.18	0.15

DCT, Dried cassava top; Ctrl, TMR contains 0% DCT; 15% DCT, TMR contains 15% DCT; 30% DCT, TMR contains 30% DCT; DM, dry matter; OM, organic matter; CP, crude protein; EE, ether extract; NSC, non-structural carbohydrate; NDF, neutral detergent fiber; ADF, acid detergent fiber; TDN, total digestible nutrient; GBP, Pound sterling.

^a^Each 1 kg of premix contains 10^6 ^IU Vit. A, 2 × 10^6^ IU Vit. D_3_, 2 × 10^4^ IU Vit. E, 130 mg Se, 8 gm Mn, 6.4 gm Zn, 10 gm Fe, 2 gm Cu, 400 mg Co, 400 mg I, 26 gm Mg, 285 mg feed preservative substances, and 10 gm feed additives (Zagromix Cattle Premix, Ltd.).

ME, MJ/kg DM = 0.157 GP + 0.084 CP + 0.22 EE − 0.081 CA + 1.06

where GP, CP, EE, and CA are total gas volume (ml), CP (%), EE (%), and ash (%), respectively.

Three inoculum bottles were sampled from each treatment after 24 h of incubation. The Gooch crucibles were filtered out of the contents of the bottle. The DM of the residue was weighed and then measured for ash before calculating IVDMD (%) and *in vitro* OM digestibility (IVOMD, %) according to Tilley and Terry [23] as follows:

IVDMD or IVOMD, % = (1 – wd – wb/ws) × 100

where wr = weight of DM or OM of residue, wb = weight of DM or OM of residue from blank, and ws = weight of DM or OM of the original sample.

### Production performance and carcass characteristics of beef cattle

The growth trial was conducted during the last 120 days of feedlot cattle, from 725 kg to final body weight. The randomized complete block design was administered: 15 Brahman × Charolais crossbred steers (50%–75% Charolais blood), average 3 years old, were used by block to have five groups by their initial body weight before randomly receiving three different TMR formulas. The cattle received TMR at 2% BW during the 120-day experimental period. All ingredients except corn silage were mixed to be the concentrate, then mixed well with corn silage prior to each meal to avoid spoiled food from high moisture content. The experimental animals were arranged in individual pens with a 2.5-m width × 3 m length. NRC [24] formulated experimental diets, and feed was offered twice daily at 7:00 am and 5:00 pm with free access to fresh water. FI and ADG are the main indicators for measuring FCR and feed cost per gain (FCG). At the end of the feeding trial, four animals from each treatment, randomized to the block (initial body weight), were slaughtered by the standard process at Nong-Sung Agricultural Cooperative Limited, Mukdahan, Thailand, and chilled at 4°C for 1 week prior to the study of carcass characteristics such as warm carcass weight, percentage of warm carcass, marbling score, loin eye area, and back fat thickness. The cooperative used the USDA standard (2017) for grading carcass quality, which was related to the marbling and maturity of beef. The economic returns of fattening steers were calculated as follows:

Total cost, GBP/head v = Steer cost + Feed cost

Total income, GBP/head = Chilled carcass weight (kg) × Price of carcass (GBP/kg)

Net income, GBP/head = Total income – Total cost.

### Statistical analysis

All data were subjected to analysis of variance using the general linear model procedure according to the model of RCBD using SAS [25].

*Y_ij_* = *µ*+ *ρ_i_* + *τ_j_* + ɛ*_ij_*, where *Y_ij_* = observation from block *i*, treatment *j*, *µ* = overall mean, *ρ_i_* = the effect of being in block *i*, *τ_j_* = the effect of being in treatment *j*, ɛ*_ij_* = error.

Treatment means were statistically compared by least significant difference at a *p *< 0.05 confidence level (*p *< 0.05), while *p*-values between 0.05 and 0.10 were declared as tendencies.

## Results

### Kinetics of gas production and degradability of experimental diets

Table 2 reveals the kinetics of cumulative gas production. The gas production from the soluble fraction (a) of the 30% DCT treatment was higher than in the other groups (*p *< 0.05). The gas production from the insoluble fraction (b) and gas production rate (c) were not significantly different (*p *> 0.05) among treatments. In addition, the potential extent of gas production (P) and degradability of DCT appeared lower than corn silage in the *in vitro* trial, although there was no statistical comparison.

### Production performance

The effects of DCT in TMR on the productive performance of Charolais crossbred steers are presented in Table 3. Fed 15% DCT in the dietary treatments of the Charolais crossbred steers, the control group was better compared to the other groups, including body weight gain (BWG), ADG, and FCR (*p* < 0.05), and 30% DCT was not significantly different (*p* > 0.05). The intake of nutrients found that the CP, TDN, and ME-fed DCT were not positively affected (*p *> 0.05), but EE, NDF, and ADF were better while supplied with 30% DCT in contrast control (*p* < 0.05). However, intake of cattle in terms of kg DM per day, %BW, and gm per kg BW^0.75^, and FCG were not different between treatments (*p *< 0.05).

### Carcass characteristics

Carcass characteristics of fattening steers fed DCT in TMR are shown in Table 4. The results show that using DCT as an ingredient in TMR did not lead to a significant difference (*p *> 0.05) in the carcass characteristics of fattening steers. Although carcass characteristics were not affected by treatments, the control group tended to be better than the DCT groups. The marbling score, which helps determine the price of the carcass, in steers fed TMR that contained DCT, was similar to the control (*p *> 0.05).

**Table 2.** *In vitro *gas production and digestibility of experimental diets.

Parameter	Corn silage	DCT	Ctrl	15% DCT	30% DCT	SEM	*p-*value
a	−4.26	−0.57	−5.99^a^	−4.05^ab^	−1.91^b^	0.43	0.042
b	64.7	25.2	60.6	56.9	48.6	5.79	0.074
c	0.073	0.125	0.085	0.086	0.089	0.004	0.461
P	60.5	24.6	54.6	52.9	46.7	5.22	0.232
DOM, %	63.3	43.2	62.6	63.0	58.1	3.04	0.368
ME, MJ/kg DM	10.3	6.88	9.57	9.78	9.09	0.48	0.353
IVDMD, %	75.2	58.5	78.2	72.9	67.7	2.36	0.484
IVOMD, %	93.2	96.2	92.8	93.2	93.6	0.29	0.459

DCT, dried cassava top; Ctrl, TMR contains 0% DCT; 15% DCT, TMR contains 15% DCT; 30% DCT; TMR contains 30% DCT.

a, the gas production from the immediately soluble fraction; b, the gas production from the insoluble fraction; c, the gas production rate constant for the insoluble fraction; P, the potential extent of gas production (a + b).

^a,b^Means in the same row with different superscripts differ between TMR contains DCT (*p *< 0.05).

**Table 3.** Productive performance of fattening cattle fed DCT in TMR.

Characteristics	Ctrl	15% DCT	30% DCT	SEM	*p-*value
No.	5	5	5		
Initial weight, kg	721	730	723	12.1	0.638
Final weight, kg	816	770	777	13.9	0.174
BWG, kg	95.6^a^	39.9^b^	53.8^ab^	13.0	0.027
ADG, gm/day	797^a^	333^b^	448^ab^	108	0.028
DM intake					
kg DM/day	14.6	13.3	14.1	0.81	0.523
% BW	1.89	1.77	1.88	0.09	0.361
gm/kg BW^0.75^	99.8	92.9	98.4	4.95	0.214
Nutrient intake, kg DM/day					
CP	1.65	1.55	1.81	0.09	0.175
EE	0.38^a^	0.48^b^	0.64^c^	0.03	0.032
NDF	4.88^a^	5.31^a^	6.51^b^	0.30	0.018
ADF	2.12^a^	2.56^a^	3.35^b^	0.14	0.023
TDN	10.1	8.97	9.26	0.55	0.091
ME (MJ/day)	139	130	128	7.77	0.086
FCR	18.3^a^	40.1^b^	31.4^ab^	4.90	0.017
FCG, GBP/kg	5.33	6.55	5.98	0.57	0.231

Ctrl, TMR contains 0% dried cassava top; 15%DCT, TMR contains 15% dried cassava top; 30% DCT, TMR contains 30% dried cassava top; DM, dry matter; OM, organic matter; CP, crude protein; EE, ether extract; NSC, non-structural carbohydrate; NDF, neutral detergent fiber; ADF, acid detergent fiber; TDN, total digestible nutrient; ME, metabolizable energy; GBP, Pound sterling; FCR = DM intake (kg/day)/ADG (kg/day); FCG = DM intake (kg/day) × Feed cost (GBP/kg)/ADG (kg/day).

^a,b,c^Means in the same row with different superscripts differ (*p *< 0.05).

**Table 4.** Carcass characteristics of fattening steers fed DCT in TMR.

Carcass characteristics	Ctrl	15% DCT	30% DCT	SEM	*p-*value
No.	4	4	4		
Slaughter weight, kg	789	755	754	33.8	0.141
Carcass weight, kg	470	438	442	22.0	0.223
Warm carcass, %	59.5	57.7	58.6	0.95	0.287
Marbling score^a^	2.88	2.63	2.75	0.20	0.652
Loin eye area, cm^2^	10.2	8.38	9.35	0.55	0.531
Back fat thickness, cm	3.05	2.80	2.03	0.66	0.159

Ctrl, TMR contains 0% dried cassava top; 15% DCT, TMR contains 15% dried cassava top; 30% DCT, TMR contains 30% dried cassava top.

^a^Marbling scores: 2 = slight, 2.5 = small, 3 = modest, 3.5 = moderate, 4 = slightly abundant, 5 = abundant.

### Production cost and economic returns

It was found that groups of fattening steers fed TMR containing DCT were not different in total cost, carcass price, or net income when compared to the control (*p* > 0.05; Table 5). However, using DCT at 15% and 30% DM in TMR did reduce feed cost by more than 20%–30% (*p *< 0.05), while the net income of steers fed 15% DCT tended to decrease because of the low carcass price.

## Discussion

DCTs in TMR did not affect *in vitro* digestibility; however, gas production from the degradable fraction (b) tended to reduce with DCT inclusion in TMR. This may be due to lower potential gas production (P) from the incubation of DCT than from corn silage; gas production from TMR with a significant amount of DCT is also reduced. Roza et al. [26] reported that too much CT meal resulted in an imbalance of rumen-degradable protein and carbohydrate, which decreased the ruminal bacterial count so that the microbes that degrade carbohydrates during fermentation also decreased and caused DM digestibility to decrease. Morm et al. [10] revealed that the CP content increased, as did IVDMD and gas production from the immediately soluble fraction, while the protozoal population decreased when using DCT between 5% and 10%. The IVDMD of DCT at 58.46% was similar to Sudarman et al. [27], who reported that the IVDMD of CT silage was 51.49%. The IVDMD of DCT was lower than that of corn silage, possibly due to higher NDF but lower starch fractions in DCT than in corn silage. The greater rumen degradability of nonstructural carbohydrates than structural carbohydrates was stated by primary works [28]. In this study, IVDMD was not reduced when using high levels of DCT (30% DM) in TMR. Here, the findings differ from those of Giang et al. [29], who studied the effects of *Leucaena *silage in dairy steers and found that digestibility values of DM and OM of rice straw with 30% *Leucaena* silage were 64.0 and 64.3, respectively, and digestibility was higher than for steers fed rice straw only. However, high fiber could reduce rumen fermentation efficiency due to the loss of methane (CH_4_) and CO_2_ via the eructation of gas during digestion. Increased levels of starch in the diet of dairy cattle reduced the amount of CH_4_ produced per unit of estimated rumen-fermentable OM [30]. Therefore, offering excessive fiber in feedlot cattle’s diet may not meet their requirements for energy for fattening, particularly the synthesis of intramuscular fat (IMF) that is related to marbling score [31].

**Table 5.** Economic returns of fattening steers fed DCTs in TMR.

Items	Ctrl	15% DCT	30% DCT	SEM	*p-*value
No.	4	4	4		
Total cost, GBP/head	1,918 ± 309	1,861 ± 218	1,831 ± 343	-	-
Steer cost	1,533 ± 226	1,553 ± 223	1,555 ± 311	-	-
Feed cost	368^a^	291^ab^	259^b^	21.3	0.023
Income, GBP/head					
Total income	2,394	2,084	2,282	115	0.491
Income over feed	2,026	1,793	2,023	123	0.246
Net income	476	224	451	100	0.072

Ctrl, TMR contains 0% dried cassava top; 15%DCT, TMR contains 15% dried cassava top; 30% DCT, TMR contains 30% dried cassava top; GBP, Pound sterling.

^a,b^Means in the same row with different superscripts differ (*p* < 0.05).

A similar intake of cattle, although fed with different levels of DCT in TMR, indicated that DCT has good palatability [32]. The growing cattle were fed DCT at 15% and 30% in TMR. The production performance of feedlot cattle in this study, ADG (333–797 gm/day), and FCR (18.3–40.1) appeared lower than in previous works. Magrin et al. [33] reported that the ADG of 228 fattened Charolais was in the range 1.46–1.53 kg/day, in accordance with Laorodphan and Likittrakulwong [34], who reported that the ADG of crossbred Charolais fed banana peel silage (1.43 kg/day) was greater than that of Napier Pakchong 1 silage (1.08 kg/day). However, crossbred Charolais × (Brahman × Thai Native) steers presented a maximum ADG of 0.56 kg/day, as reported by Thiwaratkoon et al. [35]. This should be due to differences in initial body weight, which are related to the age and growth stage of cattle. Initial body weights of cattle in Magrin et al. [33] and Laorodphan and Likittrakulwong [34] were 429.5 and 434 kg, respectively, while Thiwaratkoon et al. [35] and the present study were 609 and 720.6–730.2 kg, respectively. Feedlot entry age (111–371 days) and body weight (170.4–376.4 kg) had a highly significant impact on ADG (1.21–1.22 kg/day) of both feedlot bulls and steers (Angus × Simmental crossbred, [36]). In addition, the wide range of values of growth performance, especially ADG and FCR, in this study should also be related to growth stage, age, and initial body weight. The final stage of feedlot cattle, late 4 months, is for IMF deposition rather than increasing body weight [37]. However, other uncontrollable factors may also be affected, such as the breed of the mother and sire, nutritional management during the growing stage, and individual behavior.

The previous work [32] found Charolais cattle fed TMR containing DCT at 15% DM showed the greatest production performance and reduced feed cost (up to 15%) when compared with the control and 30% DCT inclusion. Differences in results between the current study and [32] may have been caused by nonfibrous carbohydrate content in diets that decreased with increasing DCT in TMR, resulting in increased energy (ME) intake. Nonfibrous carbohydrate content in the TMR of the previous study was 42.8% and 39.0% DM, higher than in the present study, which was 38.3% and 28.1% when 15% and 30% DCT were included, respectively. Boonsaen et al. [38] found that cassava chips provided a higher rumen-degradable carbohydrate for use as an energy source in a TMR for Kamphaeng Saen (25% Thai native × 25% Brahman × 50% Charolaise) feedlot cattle than cassava chips plus ground corn. Fermentable energy supply is usually the first limiting factor for microbial growth in the rumen; therefore, the synthesis of microbial protein in the rumen can be improved by varying the source and degradability of energy incorporated into the diet [39].

Although the present study did not find any differences in carcass characteristics, the low nonfibrous carbohydrate content in DCT may be related to a lower TDN intake by these steers than in the control group. Park et al. [37] reported that F1 Angus × Chinese Xiangxi yellow crossbred steers receiving a high-energy diet had greater longissimus dorsi muscle and IMF content than steers fed a low-energy diet, whereas dietary protein levels did not affect IMF content. High dietary levels of NFC increased ruminal concentrations of total volatile fatty acids, especially propionate [40], which is a major precursor of glucose and fatty acid synthesis [41]. On the other hand, IMF deposition can result from balancing the uptake, synthesis, and degradation of triglycerides [42]. Therefore, nonstructural and structural carbohydrates in the rumen as well as lipid addition, have to be closely considered to increase IMF deposition with appropriate production performance and cost.

Lower intake of steer-fed TMR containing DCT resulted in a low growth rate, while carcass traits that were not altered must have been the major reason for the economic return. However, Seo et al. [43] reported a high correlation between live weight and carcass percentage, and carcass grades, meaning more income from heavy cattle than from light cattle. In addition, yield grade followed a quadratic upward pattern as hot carcass weight increased but not the marbling score [44]. Income in the present study may have also been affected by the carcass grading system of the buyer (Nong-Sung Agricultural Cooperative Limited), especially the marbling score by the committee, which may be biased.

## Conclusion

In conclusion, the production performance and carcass yield of Charolais crossbred steers that received 30% DCT in TMR were similar to those of the control (no DCT), although voluntary feed intake and income tended to decrease in the 15% DCT-fed TMR group. However, in terms of utilization of local feedstuffs, especially crop residue as a sustainable feed source, CT could be a future discovery strategy for use in high-production ruminants like feedlot cattle in the present study and/or dairy cows. To optimally use DCT in TMR, balancing or increasing the energy content (particularly the easily rumen-degradable carbohydrate proportion) in feed formulations must be considered.

## List of abbreviations

CT, cassava tops; DCT, dried cassava top; FI, feed intake; HCN, hydrocyanic acid; NFC, non-fiber carbohydrate; RCBD, randomized complete block design; TDN, total digestible nutrient; TMR, total mixed ration.
